# Investigation of Key Genes and Pathways in Inhibition of Oxycodone on Vincristine-Induced Microglia Activation by Using Bioinformatics Analysis

**DOI:** 10.1155/2019/3521746

**Published:** 2019-02-10

**Authors:** Wei Liu, Jishi Ye, Hong Yan

**Affiliations:** ^1^Department of Anesthesiology, the Central Hospital of Wuhan, Tongji Medical College, Huazhong University of Science and Technology, Wuhan 430014, China; ^2^Department of Anesthesiology, Renmin Hospital of Wuhan University, Wuhan, 430060 Hubei, China

## Abstract

**Introduction:**

The neurobiological mechanisms underlying the chemotherapy-induced neuropathic pain are only partially understood. Among them, microglia activation was identified as the key component of neuropathic pain. The aim of this study was to identify differentially expressed genes (DEGs) and pathways associated with vincristine-induced neuropathic pain by using bioinformatics analysis and observe the effects of oxycodone on these DEG expressions in a vincristine-induced microglia activation model.

**Methods:**

Based on microarray profile GSE53897, we identified DEGs between vincristine-induced neuropathic pain rats and the control group. Using the ToppGene database, the prioritization DEGs were screened and performed by gene ontology (GO) and signaling pathway enrichment. A protein-protein interaction (PPI) network was used to explore the relationship among DEGs. Then, we built the vincristine-induced microglia activation model and detected several DEG expressions by real-time polymerase chain reaction (PCR) and western blotting. Meanwhile, the effects of different concentrations of oxycodone on inflammatory response in primary microglia induced by vincristine were observed.

**Results:**

A total of 38 genes were differentially expressed between normal and vincristine-treated rats. GO and pathway enrichment analysis showed that prioritization DEGs are involved in cAMP metabolic process, inflammatory response, regulation of cell proliferation, and chemokine pathway. The in vitro studies showed that vincristine had dose-dependent cytotoxic effects in microglia. Compared to the control group, vincristine (0.001 *μ*g/ml) could lead to inflammation in primary microglia induced by vincristine and upregulated the CXCL10, CXCL9, SFRP2, and PF4 mRNA and made an obvious reduction in IRF7 mRNA. At protein levels, oxycodone (50, 100 ng/ml) decreased the expression of CXCL10 and CXCL9 in activated microglia.

**Conclusion:**

Our study obtained several DEG expressions and signaling pathways in the vincristine-induced neuropathic pain rat model by bioinformatics analysis. Oxycodone could alleviate the vincristine-induced inflammatory signaling in primary microglia and downregulate some DEGs. Further molecular mechanisms need to be explored in the future.

## 1. Introduction

Neuropathic pain is identified as pain caused by a lesion or disease of the somatosensory system. A comprehensive literature search and systematic review showed that the prevalence of neuropathic pain is likely to lie between 6.9% and 10% [1, 2]. However, the current pharmacotherapy of neuropathic pain and outcome are often unsatisfactory. One of the reasons for this dilemma is the poor understanding of the pathogenesis and mechanisms for neuropathic pain. Therefore, investigation of the new therapeutic target gene and pathway for neuropathic pain seems necessary and urgent.

Chemotherapy-induced neuropathic pain is one subtype of neuropathic pain. Vincristine is a common antitumor drug, which was used to treat a mass of cancers, such as acute lymphoblastic leukemia and malignant lymphoma [3]. Nonetheless, its peripheral neurotoxicity and subsequent neuropathic pain in patients should be noted. Some animal models showed that vincristine-induced neuropathic pain may be involved in microglia activation in the dorsal root ganglia or brain [4–6]. Hence, regulation of the microglia activation induced by vincristine is a potential therapeutic direction.

Oxycodone is a *μ* and *κ* opioid receptor agonist, which was used in chronic pain, postoperative pain, visceral pain, and cancer pain [7, 8]. Emerging studies showed that oxycodone is still effective against neuropathic pain and could ameliorate the negative impact of pain on emotion and sleep [9–11]. Considering that the analgesic mechanism of oxycodone on chemotherapy-induced neuropathic pain is not clear, identifying the key molecular and pathway changes in vincristine-induced neuropathic pain remains meaningful.

In recent years, plenty of differentially expressed genes (DEGs) on neuropathic pain were identified by gene expression profiling using a microarray chip. In our study, we rerun the GSE53897 data, which was submitted by Karine et al. and stored in the Gene Expression Omnibus (GEO) database. Using bioinformatics software, we found several DEGs and key signaling pathways between the control group and the vincristine-induced neuropathic pain group. Then, we verified these DEGs in a vincristine-induced microglia activation model and observed the effects of oxycodone on these DEG expressions, hoping that these studies could further understand the neuropathic pain mechanism and analgesic mechanism of oxycodone at the molecular level.

## 2. Materials and Methods

### 2.1. Microarray Data Collection

We collected the microarray profile GSE53897 from the GEO database (http://www.ncbi.nlm.nih.gov/geo/) and performed related bioinformatics analysis in 23 September 2018. GSE53897 was based on the Illumina GPL6101 platform Illumina ratRef-12 v1.0 expression beadchip. The microarray data were chosen from the vincristine-treated rats (*n* = 4) or saline rats (*n* = 4).

### 2.2. Identification of the DEGs and Prioritization

Using a GEO2R (https://www.ncbi.nlm.nih.gov/geo/geo2r/) tool, we identified DEGs between vincristine-treated rat samples and normal samples. The cutoff criterion included the adjusted *P* value < 0.05 and ∣logFC∣ ≥ 0.5. Then, using the ToppGene database (http://toppgene.cchmc.org) with the threshold of *P* < 0.05, we evaluated whether the DEGs from GEO dataset analysis might be involved in neuropathic pain. The training gene set was obtained from GeneCards (http://genecards.org) by searching for the keywords “neuropathic pain.” The test gene set was from GSE53897 by the GEO2R tool. Then, the ToppGene database could detect the prioritized DEGs from these two sets.

### 2.3. Gene Ontology and Pathway Enrichment Analysis of the DEGs

Using the DAVID database version 6.7 (http://david.abcc.ncifcrf.gov/summary.jsp), the prioritized DEGs were enriched using gene ontology (GO) annotation analysis, and the signaling pathways were annotated using the Kyoto Encyclopedia of Genes and Genomes (KEGG) pathway analysis. The cutoff criterion *P* < 0.05 was considered statistically significant.

### 2.4. PPI Network and Module Analysis

To evaluate the protein-protein interaction (PPI), we used the STRING (version10.5) and Cytoscape (version3.7.0) tools to explore the potential relationship among those DEGs. The cutoff criterion we set included confidence score ≥ 0.4 and maximum number of interactors = 0. Moreover, we utilized the Molecular Complex Detection (MCODE) app in Cytoscape to screen modules of the PPI network. And the cutoff criteria also included degree cutoff = 2, node score cutoff = 0.2, *k* − core = 2, and max. depth = 100. Also, the top module was analyzed by GO and KEGG pathway analysis to explore the potential information.

### 2.5. Microglia Isolation, Culturing, and Activation

All procedures on animals were approved by the Experimental Animal Center Review Board of Wuhan University (Wuhan, China) and based on the US National Institutes of Health Guide for the Care. Pure neonatal microglia cultures were prepared from Sprague-Dawley rat pups. As we recently described for rat microglia, brain tissue was harvested in a sterile petri dish containing precooled phosphate-buffered solution (PBS) (Genom, Hangzhou, China), cleaned twice. Using a mesh bag (300 ml), the peeled cerebral cortex was cut up and trypsinized. After centrifugation (120*g* for 10 min), supernatant was dissected and the cells were resuspended in Dulbecco's modified Eagle's medium/nutrient mixture F-12 (DMEM/F12) medium (Gibco, New York, US). The condition of cell incubation was 37°C with 5% CO_2_. And after 48 h, the medium was changed to remove cellular debris and nonadherent cells. After 7-9 days of incubation, microglia were harvested by shaking the flasks (5 h, 65 rpm) on an orbital shaker in the incubator (37°C, 5% CO_2_). The supernatant containing microglia was collected, centrifuged, and resuspended in fresh MEM (2% FBS, 0.05 mg/ml gentamycin). After removing the supernatant, cells were resuspended in DMEM/F12 (Gibco) medium and seeded onto poly-L-lysine-coated 96-well tissue culture plates at 4^∗^10^5^ cells/cm^2^. After 1 h of incubation, the medium was changed, and the remaining cells were cultured in DMEM/F12 (Gibco) containing 10% fetal bovine serum and used as microglial cultures for cell identification following experiments.

## 3. Experimental Protocol

Microglial cells were randomly divided into the following several groups: (1) control group [the microglia in the untreated group with no vincristine (EG LABO, Boulogne-Billancourt, France) and oxycodone (Mundipharma, Beijing, China)]; (2-4) different dose vincristine groups [the microglia were treated by 0.001, 0.01, and 0.1 *μ*g/ml vincristine]; and (5-7) different dose oxycodone groups [the cells were treated with 5, 50, and 100 ng/ml oxycodone, respectively, for 1 h after the treatment of 0.001 *μ*g/ml vincristine for 24 h].

### 3.1. Cell Viability Assay

Cell Counting Kit-8 (CCK-8) assay was used to assess the effect of vincristine and/or oxycodone on cell viability assay according to the manufacturer's instructions (Beyotime, Shanghai, China). Microglial cells were seeded into 96-well plates at a density of 1^∗^10^5^ cells per well and cultured in an incubator at 37°C, 5% CO_2_ for 24 h, and then washed. CCK-8 reagent (10 *μ*l) was added to each well of different groups. 4 h later, the optical density (OD) value was measured at 450 nm using a microplate reader (model: DR-200Bs, Diatek, Wuxi, China).

### 3.2. Real-Time Polymerase Chain Reaction (RT-PCR)

The total RNA in primary microglia was isolated from the TRIzol reagent (Invitrogen™, Carlsbad, USA) according to the manufacturer's instructions. The total RNA from these samples was reverse transcribed into cDNA using a PrimeScript™ RT reagent kit with a gDNA Eraser (Takara, Kyoto, Japan). After reverse transcription, the real-time PCR was performed using SYBR Green PCR Master Mix (Takara). The following primer pairs (forward and reverse) were used: 5′-GACGGCCAGGTCATCACTATTG-3′ and 5′-AGGAAGGCTGGAAAAGAGCC-3′ for GAPDH, 5′-CCATCAGCACCATGAACCCAAGT-3′ and 5′-CACTCCAGTTAAGGAGC-3′ for CXCL10, 5′-GATCAAACCTGCCTAGATCC-3′ and 5′-GGCTGTGTAGAACACAGAGT-3′ for CXCL9, 5′-GCCCGACTTCTCCTACAAGC-3′ and 5′-TAATACGACTCACTATAGGGC-3′ for SFRP2, 5′-CGACCCGCATAAAGTGTATGAG-3′ and 5′-CCTGGTTAATGCCTGGACTTTC-3′ for IRF7, and 5′-TGCAGTGCCTGTGTGTGAAGAC-3′ and 5′-CTTCCATTCTTCAGCGTGGCTATCA-3′ for PF4. The thermocycling conditions were as follows: 40 cycles of denaturation at 95°C for 15 s, annealing at 56°C for 20 s, and extension at 72°C for 45 s. Data were calculated by the 2^-ΔΔCt^ method. GAPDH was used for the internal control. Using the NanoDrop-1000 spectrophotometer (Thermo Fisher Scientific), we detected the quality and concentration of RNA.

### 3.3. Cytokine Assay by ELISA

The culture medium supernatant was collected, and the concentration of TNF-*α* and IL-1*β* was measured by using an enzyme-linked immunosorbent assay (ELISA) kit (R&D Systems, Minneapolis, MN). The results were expressed in pg/ml.

### 3.4. Western Blot

The total protein extraction and western blot were performed as described previously. Cultured cells were collected, and cell lysates were prepared in the lysis buffer (Beyotime). The cell lysates were prepared in cell lysis buffer, and the supernatant was collected following centrifugation at 500*g* for 5 min at 4°C. Total proteins were separated by SDS-PAGE after denaturation and transferred onto polyvinylidene difluoride (PVDF) membranes. After blocking with 5% skim milk, the membranes were blocked with 5% skim milk and incubated with rabbit anti-mouse monoclonal antibodies against Cxcl10, Cxcl9, and GAPDH (Abcam, Cambridge, UK) overnight at 4°C with shaking. After washing in TBST, the membranes were exposed to the corresponding secondary horseradish peroxidase-conjugated goat anti-rabbit IgG (Santa Cruz, CA, USA) for 1.5 hours at room temperature. After washing three times, the membranes were detected using an enhanced chemiluminescence reagent kit (Aspen, Wuhan, China). All bands were scanned, and the gray values determined were analyzed using image analysis software (AlphaEaseFC software) and normalized to GAPDH.

### 3.5. Statistical Analysis

All measurement data are expressed as the mean ± standard error of the mean (SEM). The differences of mRNA and protein expressions between groups were performed by using one-way ANOVA followed by a SNK test for post hoc comparisons. All data analyses were performed by using SPSS 12.0 software. *P* < 0.05 was considered to be statistically significant.

## 4. Results

### 4.1. Identification and Prioritization for DEGs

**Figure 1:**
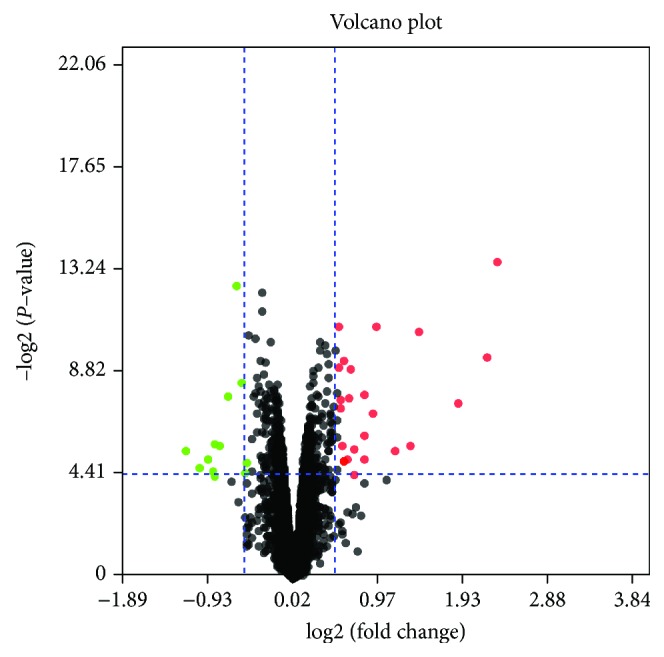
Volcano plots for DEGs. Red plots represent upregulated proteins, and green plots represent downregulated proteins. The cutoff criterion included *P* value < 0.05 and ∣logFC∣ ≥ 0.5.

Using ∣logFC∣ ≥ 0.5 and *P* value < 0.05 as cutoff criteria, a total of 38 genes were differentially expressed between normal and vincristine-treated rats, of which 25 DEGs were upregulated and 13 DEGs were downregulated (Figure 1). The training gene set was obtained from GeneCards by searching for the keywords “neuropathic pain.” According to the neuropathic pain-related genes in the GeneCards database, a total of 774 DEGs were cited and listed as the training gene, while the 38 DEGs from GSE53897 were listed as the test gene set in the ToppGene database. With the criterion *P* < 0.05, 31 candidate DEGs that might be involved in neuropathic pain were screened using the ToppGene database, of which 20 were upregulated and 11 were downregulated. All 31 candidate prioritization DEGs are summarized in Table 1.

### 4.2. GO Function Analysis and KEGG Pathway Enrichment

To further understand the 31 prioritization DEGs, GO function analysis and KEGG pathway enrichment were performed by using DAVID. As shown in Table 2, prioritization DEGs were mainly involved in biological processes (BP), including the cAMP metabolic process, cAMP-mediated signaling, leukocyte chemotaxis, cell proliferation, chemokine-mediated signaling pathway, response to lipopolysaccharide, inflammatory response, immune response, and G protein-coupled receptor signaling pathway. In terms of molecular function (MF), the DEGs were mainly enriched in CXCR3 chemokine receptor binding and chemokine activity (Table 2).

Based on KEGG pathway analysis, the 31 prioritization DEGs were identified in the chemokine signaling pathway, Toll-like receptor signaling pathway, cytokine-cytokine receptor interaction, and metabolic pathways. CXCL9, PF4, GNG11, and CXCL10 were enriched in the chemokine signaling pathway. And IRF7, CXCL9, and CXCL10 may be involved in the Toll-like receptor signaling pathway (Table 3).

### 4.3. PPI Network of DEGs and Module Analysis

**Figure 2:**
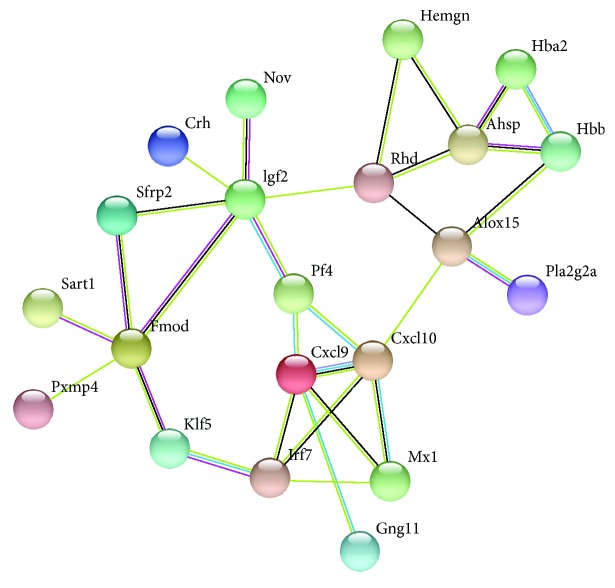
The PPI network of the screened DEGs. Network nodes represent proteins, and edges represent protein-protein associations.

**Figure 3:**
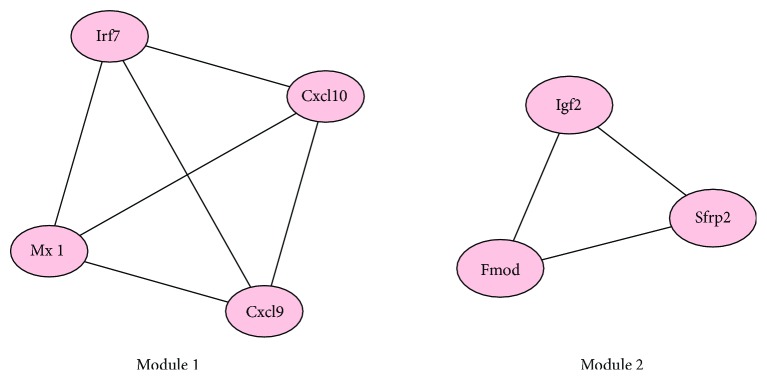
Protein module analysis in the PPI network. Using MCODE plug-in, there are 2 top modules in this network.

According to the results in the STRING multiple protein query, the PPI network of the DEGs with a higher degree of connectivity was constructed (Figure 2). There were 21 nodes and 30 edges in the network. Using MCODE plug-in, we detected significant modules in the PPI network. There are 2 top modules in this network (Figure 3). KEGG pathway enrichment analysis showed that these two modules were mainly involved in the Toll-like receptor signaling pathway, RIG-I-like receptor signaling pathway, cytosolic DNA-sensing pathway, chemokine signaling pathway, influenza A, and cytokine-cytokine receptor interaction (Figure 3, Table 4).

### 4.4. Microglial Cell Viability Test for Vincristine and/or Oxycodone Treatment

**Figure 4:**
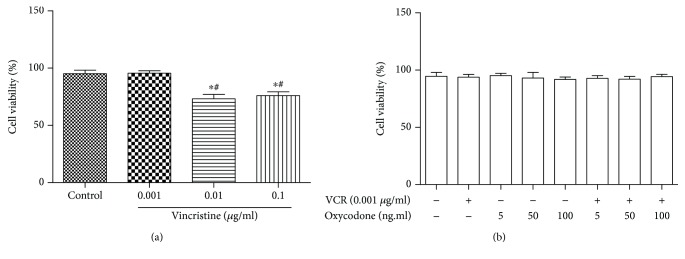
Cytotoxic effects of vincristine and/or oxycodone on primary microglia. Microglial cell viability was tested by using CCK-8 assay. Microglial cells were treated with the indicated concentration of vincristine and oxycodone. Bar charts indicate cell viability in different groups. Data are the mean ± SEM. *n* = 6. ^∗^*P* < 0.05 vs. the control group; ^#^*P* < 0.05 vs. the vincristine (0.001 *μ*g/ml) group.

Using the CCK-8 assay, cytotoxic effects of different concentrations of vincristine were tested to measure the viability of microglial cells. As shown in Figure 4, vincristine had dose-dependent cytotoxic effects in microglial cells. Compared to the control group, treatment with oxycodone (10, 50, and 100 ng/ml) and/or vincristine (0.001 *μ*g/ml) did not affect the viability of microglia (Figure 4).

### 4.5. Oxycodone Reduces the Proinflammatory Cytokine Production of Microglia Activation Induced by Vincristine

**Figure 5:**
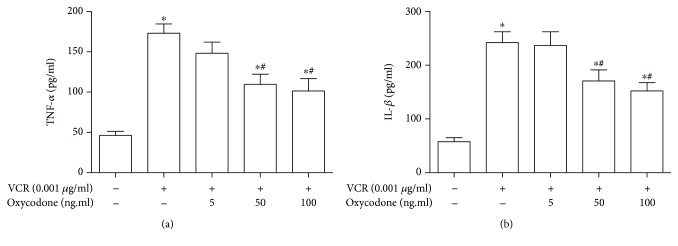
The effect of oxycodone on proinflammatory cytokine production in primary microglia induced by vincristine. (a) ELISA was performed to detect the concentration of TNF-*α* in each cultured medium. (b) ELISA was performed to detect the concentration of IL-1*β* in each cultured medium. Data are the mean ± SEM. *n* = 6. ^∗^*P* < 0.05 vs. control; ^#^*P* < 0.05 vs. the vincristine group.

Proinflammatory cytokines, such as TNF-*α* and IL-1*β*, could reflect the activation of microglia induced by vincristine. By using ELISA, we detected the concentration of vincristine-induced proinflammatory cytokines in culture supernatants. As shown in Figure 5, compared to the control group, vincristine at 0.001 *μ*g/ml could lead to microglia activation and inflammatory response, accompanied with the increased production of TNF-*α* and IL-1*β*. Low concentration of oxycodone (10 ng/ml) had no effect on the production of TNF-*α* and IL-1*β* induced by vincristine. Oxycodone at 50 and 100 ng/ml could remarkably inhibit the expression of TNF-*α* and IL-1*β* in activated microglia.

### 4.6. DEG Expression in the Vincristine-Induced Microglia Activation

**Figure 6:**
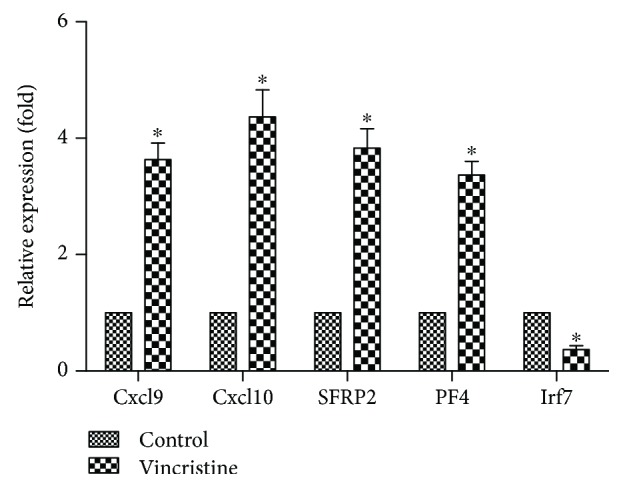
The effect of oxycodone on expression of related mRNA in primary microglia induced by vincristine. qRT-PCR was performed to test the expression of Cxcl10, Cxcl9, Irf7, SFRP2, and PF4 mRNA. And the GAPDH was used as the endogenous “housekeeping” control gene. Data are the mean ± SEM. *n* = 6. ^∗^*P* < 0.05 vs. control.

Considering the high correlation of CXCL10, CXCL9, IRF7, SFRP2, and PF4 in bioinformatics analysis by GO, KEGG enrichment, and PPI network, we then tested the mRNA expressions of the aforementioned genes in vincristine-induced microglia activation. In comparison to the control group, vincristine induced a significant increase of CXCL10, CXCL9, SFRP2, and PF4 mRNA and an obvious reduction in IRF7 mRNA in primary microglia (Figure 6).

**Figure 7:**
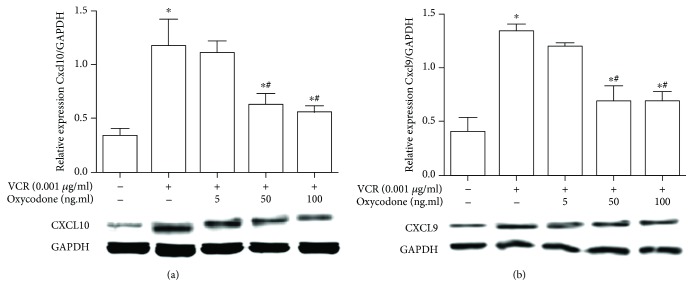
The effect of oxycodone on expression of CXCL10 and CXCL9 in primary microglia induced by vincristine. Western blot was performed to detect levels of CXCL10 and CXCL9. Bar chart demonstrated the ratio of CXCL10 and CXCL9 relative to GAPDH for each group. Data are the mean ± SEM. *n* = 6. ^∗^*P* < 0.05 vs. control; ^#^*P* < 0.05 vs. the vincristine group.

Because CXCL10 and CXCL9 had the highest correlation in the PPI network by the MCODE, we observed the expression of CXCL10 and CXCL9 at protein levels in activated microglia. As shown in Figure 7, vincristine induced a notable enhancement in CXCL10 and CXCL9 proteins in primary microglia. Low concentration of oxycodone could not reverse the increased expression. However, oxycodone at 50 and 100 ng/ml obviously decreased the expression of these genes in activated microglia.

## 5. Discussion

In this study, we screened some DEGs and signaling pathways between the control group and vincristine-induced neuropathic pain group by bioinformatics analysis. Using the vincristine-induced microglia activation model, we verified these DEG expressions and detected the effects of oxycodone on these DEG expression changes.

Considering that the DEGs are few, we lowered the standard line from ∣logFC∣ ≥ 1 to ∣logFC∣ ≥ 0.5 in GEO2R analysis. So based on these cutoff criteria, there were 38 gene DEGs between normal and vincristine-treated rats, including 25 DEGs upregulated and 13 DEGs downregulated. Utilizing the DAVID database, the gene ontology enrichment and KEGG analysis found that these prioritized DEGs are mainly involved in the chemokine signaling pathway, regulation of cell proliferation, inflammatory response, and G protein-coupled receptor signaling pathway. In fact, the relationship between the chemokine signaling pathway and the G protein-coupled receptor signaling pathway is rather close and their function is complementary. The cross talk in chemokines and G protein-coupled receptors could lead to the migration of leukocytes during normal immune function. In tissue injury or infection, this interaction is a key component of the inflammatory response [12, 13]. Current studies showed that several chemokines, such as MCP-1, fractalkine, and SDF-1, have been linked to chronic and neuropathic pain and as the key inflammatory mediators in both human conditions and animal models [14–16]. These chemokines are expected to be an attractive therapeutic target for chronic and neuropathic pain. In addition, regulation of cell proliferation was also identified in bioinformatics analysis. A series of studies have reported that astroglial and microglial cell proliferations are along with neuropathic pain and could be regulated by some cytokines and chemokines, which are potential targets for the optimal treatment of neuropathic pain [17, 18].

The PPI network of DEGs and module analysis found that CXCL10, CXCL9, IRF7, SFRP2, and PF4 have high connectivity and are valued to be focused on further. So in the vincristine-induced microglia activation model, we tested these gene expressions at mRNA levels. CXCL10 and CXCL9 are protein-coding genes and the members of the CXC subfamily and ligand for the receptor CXCR3 [19]. Related animal models showed that chemokines played an important role in the pathophysiology of neuropathic pain. Bäckryd et al. found that high levels of chemokines CXCL6, CXCL10, CCL8, CCL11, and CCL23 in cerebrospinal fluid were remarkably higher in a neuropathic pain patient compared with those in a healthy person [20]. The upregulated mRNA of CxcL10 in vincristine-induced microglia activation is coincident with the above study. However, a notable study exhibited an intriguing viewpoint. Although the expressions of CXCL9 and CXCL11 are upregulated in spinal nerve injury, Wu et al. thought that spinal CXCL9 and CXCL11 are not involved in neuropathic pain. They found that hyperalgesia or allodynia behaviors had no obvious change after intrathecal injection of CXCL9 and CXCL11. For central sensitization marker, extracellular signal-regulated protein kinases (ERKs), this intervention did not induce its activation [21]. Despite the controversy, chemokines remain the focus and potential target for neuropathic pain.

Vincristine is a common chemotherapeutic drug, which could lead to the peripheral neuropathy and neuropathic pain. In both animal models and clinical practice, this phenomenon had been demonstrated [5, 6, 22]. Although microglia activation had been found in vincristine-induced neuropathic pain in a rat model, the cell model was not validated. Our studies found that medium and high concentration of vincristine decreased the cell viability of microglia. Meanwhile, low concentration of vincristine (0.001 *μ*g/ml) both could induce microglia activation and has no effect the cell viability of microglia, which may be more in line with the actual situation. So we chose the 0.001 *μ*g/ml vincristine to build the microglia activation model. Oxycodone is a widely used analgesic due to its several advantages. Except for *μ* and *κ* receptor activation, the attenuation of the inflammatory response in microglia and the regulation of GABAB receptor are also involved in the analgesic effect of oxycodone, especially for chemotherapy-induced neuropathic pain [23, 24]. In our study, we demonstrated that oxycodone can inhibit the inflammation in the primary microglia induced by vincristine, which may be related to the reduction of CXCL10 and CXCL9 expression. Identification of the DEG alteration induced by vincristine is critical to illustrating the potential molecular mechanisms involved in the drug action and tolerance. Moreover, finding the DEGs by oxycodone treatment in vincristine-treated microglia activation affords us with more insight into the molecular mechanisms managing the sustained analgesic effect of opioids in the chemotherapy-induced neuropathic pain state.

In neuropathic pain, few studies paid attention to IRF7, SFRP2, and PF4. IRF7 is a member of the interferon regulatory transcription factor (IRF) family and has been reported to participate in the transcriptional activation of virus-inducible cellular genes, including interferon beta chain genes [25]. Although no study linked IRF7 to neuropathic pain and other members of its family, an IRF8-IRF5 transcriptional axis may contribute to microglia activation in the neuropathic pain animal model through the P2X4R pathway [26]. SFRPs are soluble modulators of Wnt signaling, which may be involved in axonal regeneration in the central nervous system and enhance locomotor recovery after spinal cord injury [27]. Diseases associated with PF4 included thrombocytopenia and megakaryocytic leukemia [28, 29]. GO analysis enriched this gene to heparin binding and CXCR3 chemokine receptor binding. Considering that the CXCR3 chemokine receptor is a hot spot in neuropathic pain, further research is needed to reveal the role of these genes in chemotherapy-induced neuropathic pain.

## 6. Conclusion

Our study suggested that there are several DEG expressions and signaling pathways in the vincristine-induced neuropathic pain rat model. Oxycodone could effectively alleviate the vincristine-induced inflammatory in primary microglia and downregulate the DEGs (Cxcl10 and Cxcl9). Further molecular biological experiments are required to make sure the function of DEGs in chemotherapy-induced neuropathic pain.

## Figures and Tables

**Table 1:** 31 candidate prioritization differentially expressed genes from the ToppGene.

Rank	Gene name	Gene symbol	Interactant count	Score	Expression
1	Hemoglobin subunit beta	HBB	63	9.22*E*-02	0.9249
2	Platelet factor 4	PF4	11	9.08*E*-02	2.1651
3	Hemoglobin subunit alpha 2	HBA2	53	7.10*E*-02	0.5341
4	Cystatin E/M	CST6	38	6.20*E*-02	-0.6299
5	Squamous cell carcinoma antigen recognized by T cell 1	SART1	119	5.18*E*-02	-0.9107
6	Insulin-like growth factor 2	IGF2	24	3.87*E*-02	0.6439
7	Nephroblastoma overexpressed	NOV	18	3.71*E*-02	0.8820
8	Kruppel-like factor 5	KLF5	54	3.58*E*-02	0.5621
9	Interferon regulatory factor 7	IRF7	48	3.26*E*-02	-0.5762
10	Mitochondrial ribosomal protein L11	MRPL11	65	3.22*E*-02	-0.8362
11	Phospholipase A2 group IIA	PLA2G2A	29	2.89*E*-02	1.3113
12	Solute carrier family 47 member 1	SLC47A1	27	2.83*E*-02	0.7889
13	Myxovirus (influenza virus) resistance	MX1	43	2.75*E*-02	-0.7250
14	SCY1-like pseudokinase 1	SCYL1	32	2.19*E*-02	-1.0549
15	C-X-C motif chemokine ligand 10	CXCL10	10	1.82*E*-02	0.9672
16	C-X-C motif chemokine ligand 9	CXCL9	9	1.42*E*-02	0.8880
17	Tektin 2	TEKT2	19	1.34*E*-02	0.5100
18	Secreted frizzled-related protein 2	SFRP2	20	1.25*E*-02	0.7842
19	Sterol-C5-desaturase	SC5D	12	1.14*E*-02	-0.5514
20	Hemogen	HEMGN	17	1.07*E*-02	0.564234
21	Ring finger protein 113A2	RNF113A	20	9.74*E*-03	-1.7287
22	Guanine nucleotide binding protein (G protein), gamma 11	GNG11	11	9.36*E*-03	0.7967
23	Troponin T2, cardiac type	TNNT2	11	7.86*E*-03	0.5085
24	Corticotropin-releasing hormone	CRH	5	5.88*E*-03	-0.8934
25	Gap junction protein, beta 6	GJB6	6	4.49*E*-03	0.5675
26	Alpha hemoglobin-stabilizing protein	AHSP	7	3.88*E*-03	1.1388
27	Fibromodulin	FMOD	7	3.14*E*-03	0.5468
28	Arachidonate 15-lipoxygenase	ALOX15	4	2.46*E*-03	3.8408
29	Peroxisomal membrane protein 4	PXMP4	1	9.57*E*-04	-1.2075
30	Cartilage intermediate layer protein	CILP	2	7.60*E*-04	0.6077
31	Rh blood group, D antigen	RHD	1	2.97*E*-04	0.5245

**Table 2:** Gene ontology analysis of differentially expressed genes.

Category	Term	Count	*P* value	Genes
GOTERM_MF_DIRECT	GO:0048248—CXCR3 chemokine receptor binding	3	2.16*E*-05	CXCL9, PF4, CXCL10
GOTERM_BP_DIRECT	GO:0030816—positive regulation of cAMP metabolic process	3	1.10*E*-04	CXCL9, PF4, CXCL10
GOTERM_BP_DIRECT	GO:0043950—positive regulation of cAMP-mediated signaling	3	2.21*E*-04	CXCL9, PF4, CXCL10
GOTERM_BP_DIRECT	GO:0002690—positive regulation of leukocyte chemotaxis	3	2.55*E*-04	CXCL9, PF4, CXCL10
GOTERM_BP_DIRECT	GO:0042127—regulation of cell proliferation	5	4.92*E*-04	SFRP2, PLA2G2A, CXCL9, PF4, CXCL10
GOTERM_MF_DIRECT	GO:0008009—chemokine activity	3	0.001322	CXCL9, PF4, CXCL10
GOTERM_BP_DIRECT	GO:0070098—chemokine-mediated signaling pathway	3	0.003717	CXCL9, PF4, CXCL10
GOTERM_BP_DIRECT	GO:0032496—response to lipopolysaccharide	4	0.009824	CXCL9, PF4, GJB6, CXCL10
GOTERM_BP_DIRECT	GO:0006954—inflammatory response	4	0.0115218	CXCL9, CRH, PF4, CXCL10
GOTERM_BP_DIRECT	GO:0006955—immune response	3	0.0673817	CXCL9, PF4, CXCL10
GOTERM_BP_DIRECT	GO:0007186—G protein-coupled receptor signaling pathway	5	0.4113867	SFRP2, CXCL9, PF4, GNG11, CXCL10
GOTERM_CC_DIRECT	GO.0005833—hemoglobin complex	2	0.3525113	Hba2, Hbb

**Table 3:** Kyoto Encyclopedia of Genes and Genomes pathway enrichment of differentially expressed genes.

Pathway name	Gene count	*P* value	Genes
rno04062: chemokine signaling pathway	4	0.005212	CXCL9, PF4, GNG11, CXCL10
rno04620: Toll-like receptor signaling pathway	3	0.0164784	IRF7, CXCL9, CXCL10
rno04060: cytokine-cytokine receptor interaction	3	0.0713334	CXCL9, PF4, CXCL10
rno01100: metabolic pathways	3	0.7716595	SC5D, ALOX15, PLA2G2A

**Table 4:** The enriched pathways of module 1.

Pathway ID #	Pathway description	Gene count	False discovery rate	Genes
4620	Toll-like receptor signaling pathway	3	6.84*E*-05	Cxcl10, Cxcl9, Irf7
4622	RIG-I-like receptor signaling pathway	2	0.00381	Cxcl10, Irf7
4623	Cytosolic DNA-sensing pathway	2	0.00381	Cxcl10, Irf7
4062	Chemokine signaling pathway	2	0.0184	Cxcl10, Cxcl9
5164	Influenza A	2	0.0184	Cxcl10, Irf7
4060	Cytokine-cytokine receptor interaction	2	0.0232	Cxcl10, Cxcl9

## Data Availability

The datasets analyzed during the current study are available from the corresponding author on reasonable request.

## References

[1] Blyth F. M. (2018). Global burden of neuropathic pain. *Pain*.

[2] Hietaharju A., Finnerup N. B. (2018). Introduction to the *PAIN* supplement on neuropathic pain. *Pain*.

[3] Wahlman C., Doyle T. M., Little J. W. (2018). Chemotherapy-induced pain is promoted by enhanced spinal adenosine kinase levels through astrocyte-dependent mechanisms. *Pain*.

[4] Jaggi A. S., Kaur G., Bali A., Singh N. (2017). Pharmacological investigations on mast cell stabilizer and histamine receptor antagonists in vincristine-induced neuropathic pain. *Naunyn-Schmiedeberg's Archives of Pharmacology*.

[5] Chiba T., Oka Y., Sashida H. (2017). Vincristine-induced peripheral neuropathic pain and expression of transient receptor potential vanilloid 1 in rat. *Journal of Pharmacological Sciences*.

[6] Gong S. S., Li Y. X., Zhang M. T. (2016). Neuroprotective effect of matrine in mouse model of vincristine-induced neuropathic pain. *Neurochemical Research*.

[7] Gaskell H., Derry S., Stannard C., Moore R. A., Cochrane Pain, Palliative and Supportive Care Group (2016). Oxycodone for neuropathic pain in adults. *Cochrane Database of Systematic Reviews*.

[8] Davis M. P., Goforth H. W. (2016). Oxycodone with an opioid receptor antagonist: a review. *Journal of Opioid Management*.

[9] Gavin P. D., Tremper L., Smith A., Williams G., Brooker C. (2017). Transdermal oxycodone patch for the treatment of postherpetic neuralgia: a randomized, double-blind, controlled trial. *Pain Management*.

[10] Thorn D. A., Zhang Y., Li J. X. (2017). Tolerance and cross-tolerance to the antinociceptive effects of oxycodone and the imidazoline I_2_ receptor agonist phenyzoline in adult male rats. *Psychopharmacology*.

[11] Gaspari S., Cogliani V., Manouras L. (2017). RGS9-2 modulates responses to oxycodone in pain-free and chronic pain states. *Neuropsychopharmacology*.

[12] Hughes C. E., Nibbs R. J. B. (2018). A guide to chemokines and their receptors. *The FEBS Journal*.

[13] Vanheule V., Crijns H., Poosti F. (2018). Anti-inflammatory effects of the GAG-binding CXCL9 (74-103) peptide in dinitrofluorobenzene-induced contact hypersensitivity in mice. *Clinical and Experimental Allergy*.

[14] Luo X., Tai W. L., Sun L. (2016). Crosstalk between astrocytic CXCL12 and microglial CXCR4 contributes to the development of neuropathic pain. *Molecular Pain*.

[15] Zhang Z. J., Jiang B. C., Gao Y. J. (2017). Chemokines in neuron-glial cell interaction and pathogenesis of neuropathic pain. *Cellular and Molecular Life Sciences*.

[16] Gu N., Peng J., Murugan M. (2016). Spinal microgliosis due to resident microglial proliferation is required for pain hypersensitivity after peripheral nerve injury. *Cell Reports*.

[17] Ke B. C., Huang X. X., Li Y. (2016). Neuronal-derived Ccl7 drives neuropathic pain by promoting astrocyte proliferation. *Neuroreport*.

[18] Fernández-Martos C. M., González P., Rodriguez F. J. (2012). Acute leptin treatment enhances functional recovery after spinal cord injury. *PLoS One*.

[19] Carr F. B., Géranten S. M., Hunt S. P. (2014). Descending controls modulate inflammatory joint pain and regulate CXC chemokine and iNOS expression in the dorsal horn. *Molecular Pain*.

[20] Bäckryd E., Lind A. L., Thulin M., Larsson A., Gerdle B., Gordh T. (2017). High levels of cerebrospinal fluid chemokines point to the presence of neuroinflammation in peripheral neuropathic pain: a cross-sectional study of 2 cohorts of patients compared with healthy controls. *Pain*.

[21] Wu X. B., He L. N., Jiang B. C. (2018). Spinal CXCL9 and CXCL11 are not involved in neuropathic pain despite an upregulation in the spinal cord following spinal nerve injury. *Molecular Pain*.

[22] Madden K., Bruera E. (2017). Very-low-dose methadone to treat refractory neuropathic pain in children with cancer. *Journal of Palliative Medicine*.

[23] Thibault K., Calvino B., Rivals I. (2014). Molecular mechanisms underlying the enhanced analgesic effect of oxycodone compared to morphine in chemotherapy-induced neuropathic pain. *PLoS One*.

[24] Ye J., Yan H., Xia Z. (2018). Oxycodone ameliorates the inflammatory response induced by lipopolysaccharide in primary microglia. *Journal of Pain Research*.

[25] Chinnaswamy S., Bhushan A., Behera A. K. (2016). Roles for transcription factors Sp1, NF-*κ*B, IRF3, and IRF7 in expression of the human *IFNL4* gene. *Viral Immunology*.

[26] Masuda T., Iwamoto S., Yoshinaga R. (2014). Transcription factor IRF5 drives P2X4R^+^-reactive microglia gating neuropathic pain. *Nature Communications*.

[27] Perumal V., Dharmarajan A. M., Fox S. A. (2017). The Wnt regulator SFRP4 inhibits mesothelioma cell proliferation, migration, and antagonizes Wnt3a via its netrin-like domain. *International Journal of Oncology*.

[28] Miyachi H., Reinhardt J. W., Otsuru S. (2018). Bone marrow-derived mononuclear cell seeded bioresorbable vascular graft improves acute graft patency by inhibiting thrombus formation via platelet adhesion. *International Journal of Cardiology*.

[29] Sain M., Burilovic V., Tomicic M., Jelicic I. (2018). High ANTI-PF4/heparin antibodies titer and thromboses due to infection 9 months after cessation of heparin in hemodialyzed patient with heparin-induced thrombocytopenia. *Therapeutic Apheresis and Dialysis*.

